# Lung Cancer in Homeless People: Clinical Outcomes and Cost Analysis in a Single Institute

**DOI:** 10.1155/2016/3727689

**Published:** 2016-04-27

**Authors:** Koung Jin Suh, Ki Hwan Kim, Jin Lim, Jin Hyun Park, Jin-Soo Kim, In Sil Choi

**Affiliations:** Department of Internal Medicine, Seoul National University Boramae Medical Center, Seoul, Republic of Korea

## Abstract

*Introduction*. To characterize the demographic and clinical features, outcomes, and treatment costs of lung cancer in homeless people.* Methods*. Medical records of 22 homeless patients with lung cancer at Seoul National University Boramae Medical Center in Seoul, South Korea, were retrospectively analyzed.* Results*. All patients were men (median age, 62 years). Most patients (78%) had advanced disease (stage IIIB, *n* = 2; stage IV, *n* = 15). Seven died during initial hospitalization (median survival, 1.5 months). Six were lost to follow-up after initial outpatient visits or discharges from initial admission (median follow-up, 13 days). Only 4 received appropriate treatment for their disease and survived for 1, 15, 19, and 28 months, respectively. Conversely, 4 of 5 patients with early stage disease (stage I, *n* = 4; stage IIA, *n* = 1) received curative surgery (median follow-up 25.5 months). The median treatment cost based on 29 days of hospitalization and 2 outpatient visits was $12,513, constituting 47.3% of the 2013 per capita income. Inpatient treatment accounted for 90% of the total costs. The National Health Insurance Service paid 82% of the costs.* Conclusion*. Among the homeless, lung cancer seems to be associated with poor prognosis and substantial costs during a relatively short follow-up and survival period.

## 1. Introduction

Lung cancer is the leading cause of cancer death in the world, accounting for 22.2% of all cancer deaths [1, 2]. The primary risk factor, causing approximately 85–90% of lung cancers, is cigarette smoking [3]. Homeless people have a higher prevalence of smoking [4] and are therefore at high risk for developing lung cancer. Indeed, cancer is among the leading causes of death among homeless adults with neoplasms of the trachea, bronchus, and lungs, comprising over one-third of all cancer deaths [5, 6].

Despite their high cancer burden, homeless people have limited access to medical care and therefore experience increased mortality compared to the general population [5, 7]. Barriers to medical services include cost, transportation, competing needs, psychiatric illness, and medical provider bias [8, 9]. Cancer screening rates are also lower in the homeless population than in the general population [4, 10, 11]. Therefore, homeless people are more likely to be diagnosed with advanced stage lung cancer. Late stage diagnosis limits curative treatment for the homeless and also puts a substantial economic burden on central and/or local governments.

Numerous studies have examined cancer related risk factors, screening frequencies, and mortality in the homeless. However, little is known about disease stage at diagnosis, treatment strategies, clinical outcomes, and related costs in this vulnerable population. Comprehensive understanding of these issues will help define the most urgent needs of the homeless and allow development of approaches to meet those needs. Herein, we analyzed the demographic and clinical features, outcomes, and treatment costs of homeless adults with lung cancer.

## 2. Methods

### 2.1. Study Design and Patient Population

A retrospective study of patients diagnosed with lung cancer at a single secondary referral hospital in South Korea was conducted. In Seoul, with a population of 12 million, there are 8 public hospitals designated to treat the homeless, one of which is the 800-bed Seoul National University Boramae Medical Center (SNU-BMC). SNU-BMC has a homeless-patient ward (30 beds) and a homeless-patient care unit in the emergency department.

Costs incurred specifically for lung cancer from diagnosis through the subsequent 2 years were calculated. Patients newly diagnosed with lung cancer at the SNU-BMC between January 2005 and March 2014 were eligible for inclusion. Patients with a registered residence were excluded. The Institutional Review Board at Seoul National University Boramae Medical Center approved this study (IRB number 20140128/26-2014-9/022), and it was conducted in accordance with the Declaration of Helsinki. Written informed consent was not acquired because it was a retrospective study. All patients' records/information were anonymized and deidentified prior to analysis.

### 2.2. Cost Definitions and Data Sources

Costs consisted of the following 3 components: inpatient costs, outpatient costs, and prescription drug costs. Inpatient and outpatient cost information was retrieved from the institutional accounting system. Medical costs from emergency room visits were inpatient costs. Costs of prescription drugs sold in the hospital pharmacy were included in the cost analysis. However, costs of drugs bought outside the hospital could not be included owing to insufficient data. The proportion of medical expenses not covered by the National Health Insurance Service (NHIS) was determined, and the institutions financially supporting these expenses were identified. All cost estimates are represented according to the annual exchange rate in 2014 (1060 Korean Won = 1 US dollar).

Demographic and disease data collected included age, sex, pathologic type, clinical and pathologic stage, recurrence, number of outpatient visits, and hospitalization duration. Advanced stage disease was defined as stage IIIB or stage IV. All other stages were defined as early stage disease. The follow-up period was defined from the date of diagnosis to the end of follow-up at our institution. The overall survival (OS) was defined from the date of diagnosis to death. The date of death was determined from medical records at SNU-BMC, or for cases lost to follow-up at our institution, from the Statistics Korea (KOSTAT) database by using resident registration numbers.

### 2.3. Data Analysis

Total costs were compared between patients with early and advanced disease by using the Mann-Whitney test. *P* values of < 0.05 were considered significant. OS was calculated by using the Kaplan-Meier method, and the values were compared by using the log-rank test. All analyses of data collected through February 2015 were performed by using SPSS software version 21.

## 3. Results

### 3.1. Patient Characteristics

Characteristics of the 22 homeless patients diagnosed with lung cancer are summarized in Table 1. All patients were men, with a median age of 62 years. Fifteen patients (68%) were current smokers, 9 patients (40.9%) had a history of alcohol abuse, and 4 patients (18%) had mental illnesses including schizophrenia and dementia. Most patients (78%) presented with advanced disease. Homeless status was different between the advanced and early stage patients; seven of the patients with advanced disease lived on the streets, whereas none of the patients with early stage disease lived on the streets. None of the patients underwent tests for EGFR mutation, KRAS mutation, or ALK gene rearrangement, although 9 of the patients had non-small cell lung cancer of nonsquamous histology.

### 3.2. Clinical Outcomes

**Figure 1:**
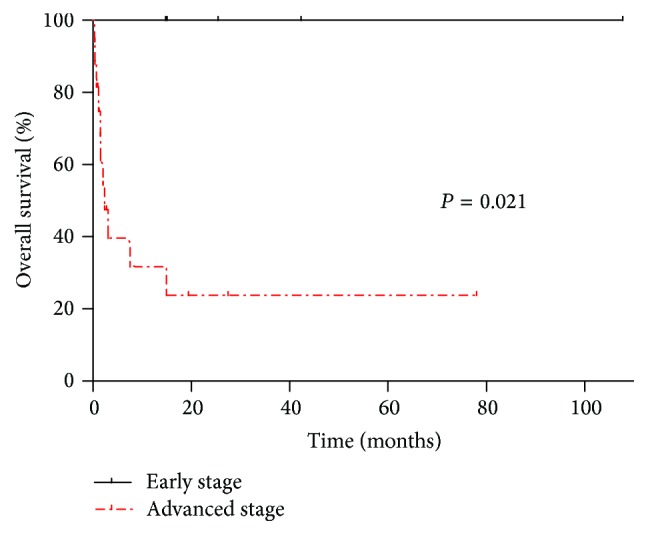
The probability of overall survival for early and advanced stage lung cancer.

The median follow-up duration and estimated OS for all patients were 1.8 months (range, 0–107.7) and 7.5 months (95% confidence intervals [CI], 0–30.2), respectively. As expected, patients with advanced disease had poorer outcomes than those with early stage disease. The median follow-up duration was 1.1 months (range, 0–19.4) for advanced disease compared to 25.5 months (range, 14.7–107.7) for early stage disease. The estimated median OS for the advanced stage group was 2.3 months (95% CI, 0.6–4.1), and the median OS for the early stage group was not calculated (*P* = 0.013) (Figure 1).

### 3.3. Advanced Disease

Of the 17 patients with advanced disease, 7 (41%) died during initial hospitalization (median survival, 1.5 months; range, 0.4–3.1). Of these, 2 were admitted to the intensive care unit and died after cardiopulmonary resuscitation without discussing a terminal care plan. Six patients (35%) were lost to follow-up after an initial visit or discharge from the initial admission (median follow-up, 13 days; range, 1–30). Using KOSTAT database, we determined that 3 of these patients had died by the time of analysis, with an estimated median survival of 7.7 months (95% CI, 1.9–13.4). Only 4 patients (24%) received appropriate treatment for their lung cancer and/or related symptoms, with a median follow-up duration of 13.7 months (range, 1.2–19.4). Of these, 2 received chemotherapy and/or tyrosine kinase inhibitors; both eventually showed disease progression, and 1 patient subsequently died. One patient underwent craniotomy for tumor resection, and the final patient received whole-brain radiation therapy. They survived for 1, 15, 19, and 28 months, respectively.

### 3.4. Early Stage Disease

Four out of the 5 patients with early stage disease received curative surgery. One patient was inoperable owing to poor lung function. The median follow-up was 25.5 months (range, 14.7–107.7), and none of the patients who received surgery showed recurrence or died.

### 3.5. Costs, Days of Hospitalization, and the Number of Outpatient Clinic Visits

One of the 5 patients with early stage disease and 4 of the 17 patients with advanced stage disease were excluded in the cost analysis owing to lack of data in the institutional accounting system. Three additional patients were excluded because they were lost to follow-up after only one outpatient visit. Cost analysis was performed for the remaining 14 patients (Table 2).

The median length and the cost of initial hospitalization were 29 days (range, 7–67) and $8,619 (range, $2,925–$27,839), respectively. The median number of outpatient visit was 2 (range, 0–33), with a cost of $949 (range, $19–$5,916). The median length and the costs of further hospitalization were not calculated, but the mean length and costs of further hospitalization were 5 days (range, 0–26) and $1,083 (range, $0–$6,435), respectively. Considering 29 days of hospitalization and 2 outpatient visits, the median treatment cost was $12,513, constituting 47.3% of the per capita income in 2013. The majority of the costs (90%) were attributed to inpatient treatment. The NHIS paid 82% of the costs, and the remainder costs were financed by the homeless shelter in which the patient lived or by the Seoul Metropolitan Government if the patient lived on the streets.

### 3.6. Cost Comparison between Early Stage and Advanced Stage Disease

The length of hospitalization was similar for patients with early and advanced stage diseases (34 days for both groups), but the number of outpatient visits was higher in the early stage group (11 versus 0) (Table 3). The median costs for admission and outpatient visits were higher in the early stage group than in the advanced stage group ($12,567 and $1,274 compared to $8,619 and $1,146, resp.). The total costs for the early stage and advanced stage groups were $14,153 and $11,100, respectively. Considering higher mortality in the advanced stage group, we also calculated the cost per day considering the number of days of follow-up. The median cost per day followed-up was $138 for early stage patients and $255 for advanced stage patients. None of the variables including the number of visits and the costs were significantly different between the two groups.

**Figure 2:**
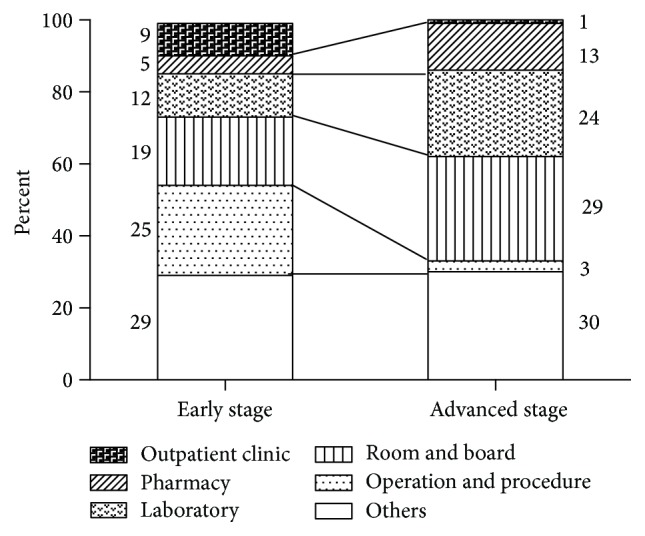
Categories of cost for lung cancer treatment according to the stage. The contribution of each category to total cost is represented as percentage values.

The categories of cost for different stages of disease are summarized in Figure 2. The major contributors to costs were room and board and laboratory services for both groups. However, the proportion of categories contributing to costs was different. Advanced stage patients had relatively longer hospitalization durations, despite short follow-up and survival, with costs for room and board and medicines as the main contributors. Patients with early stage disease tended to have surgery and related procedures, with the costs for these services as the main contributors.

## 4. Discussion

In this study of a small sample of homeless men, most homeless patients (78%) were diagnosed with advanced disease; distant metastases were present in 69% of these patients. Although 57% of patients with lung cancer are diagnosed with distant metastasis in the general population, the proportion of advanced disease at diagnosis is higher in this homeless sample [12]. The devastating consequence of late diagnosis is evident in patient survival. Most of the homeless patients with advanced cancer in our study (76%) died shortly after diagnosis or were lost to follow-up with a median OS of only 2.4 months. In contrast, most patients diagnosed with early stage disease underwent curative surgery; their survival was comparable with that of the general population. The prevalence of smoking was much higher in our homeless sample (68%) than in the Korean male population in 2010 (43.7%) [13]. Lung cancer, if diagnosed at an early stage, is potentially curable via surgical resection. Therefore, the higher smoking rate and late diagnosis suggest that primary prevention and cancer screening in this high-risk group are urgently needed.

Many studies have shown that populations with low socioeconomic status, including the homeless, undergo less cancer screening and are diagnosed with advanced stage disease [4, 10, 11, 14, 15]. In a survey of 221 homeless adults, >75% of the respondents believed in the benefits of screening; a majority of respondents (63%) believed that if a cancer is detected early, it is likely to be cured. Despite this accurate knowledge of cancer screening guidelines, screening rates by using endoscopy and the prostate specific antigen test were lower in these homeless patients than in the general population [4].

Even if an adequate screening program for the homeless is provided, adherence to screening guidelines and proper follow-up is difficult. However, studies have shown that, given the opportunity, homeless people are willing to obtain health care for chronic conditions when they believe such care to be important [16]. Moore and Durden conducted head and neck cancer screening and education for a homeless population (*n* = 300), with 80% of the homeless participants appearing for follow-up at a designated facility for further evaluation and treatment [17]. Of these, 9% were diagnosed with cancer and treated. In our study, all the early stage disease patients were regularly followed up and successfully treated. Therefore, proper cancer screening services should be provided to this neglected population.

Hospitalization costs associated with admission of homeless patients with increased length of stay are known to be high [18] and may present a substantial burden on the resources of safety-net hospitals [19]. In our study, the median cost for treatment of homeless lung cancer patients was substantial, and a large proportion of these costs (82%) were paid by the NHIS, with the remainder paid by the homeless shelter and/or the local government. Costs were higher in the early stage group. However, considering that patients with early stage disease underwent curative surgery and had longer follow-up and survival, the similar cost between both the groups is encouraging.

This study also emphasizes the importance of palliative care for the homeless population, another aspect which has not been previously addressed. In our study, 41% of the patients with advanced disease died within 2 months of diagnosis, and some of them were admitted to the intensive care unit and died after cardiopulmonary resuscitation, which was an effort to no purpose. More than half of lung cancer patients are diagnosed at an advanced stage, and a substantial proportion of these patients are terminally ill. Most homeless patients live in environments not suitable for terminal care, and many are found dead in public places, or they die in emergency departments without any appropriate pain and symptom management [20]. Therefore, it is important to discuss the end-of-life issues including do-not-resuscitate orders from the time of diagnosis and to provide palliative care. A study of shelter-based palliative care offers unique insight [21]. Twenty-eight terminally ill homeless patients were admitted to a 15-bed shelter-based palliative care unit with a full-time registered nurse and a client care worker, where they received pain and symptom management through consultation with a specialist physician collaborating with the project. These patients received support and spiritual care and died under hospice care surrounded by family and friends. Furthermore, compared to alternate locations of terminal care such as tertiary care hospitals, the average health care cost per admission to a hospice program was significantly lower ($15,000 versus $64,600, *P* < 0.001). In this regard, the model of shelter-based medical care seems very promising for effective delivery of screening program and palliative care.

This study has several limitations. First, we focused on adults who were diagnosed and treated at the SNU-BMC in Seoul. Our findings may not be generalizable to homeless populations treated at other hospitals or in other cities. However, approximately half the homeless population in Korea is in Seoul, and the SNU-BMC is the second largest of the 8 hospitals designated for the treatment of homeless populations. Therefore, the sample in our study should be representative of the homeless population in Korea. Second, this was a retrospective study with a small sample size. Important information such as duration of homelessness and duration of smoking was not always documented. Also, due to extremely small sample size of the early stage group, none of the comparisons regarding the number of visits and the treatment costs between the stages at diagnosis reached statistical significance although the numerical difference was noticed. Third, we calculated costs related to hospital admission and outpatient visits from the SNU-BMC accounting system, but patients may have visited other hospitals and incurred costs related to their lung cancer, which were not captured. Therefore, the actual costs may be higher. Moreover, owing to different health care systems as well as different reimbursement policies and monetary values, direct cost comparison with other countries is difficult.

Despite these limitations, this study is the first attempt to better understand the demographic and clinical outcomes of homeless lung cancer patients, as well as to estimate treatment costs. For the early stage group, being homeless did not prevent the receipt of appropriate treatment and follow-up, with clinical outcomes comparable to the general population. Patients with advanced stage lung cancer, constituting the majority of the cases, had dismal prognosis. The costs of lung cancer treatment for homeless individuals were substantial, presenting a burden on the national and local governments. Therefore, proper screening programs and specialized palliative care for this high-risk population are urgently needed.

## Figures and Tables

**Table 1:** Patient characteristics (*n* = 22).

Subjects	*n* (%)
Median age (range), years	62 (27–88)
Sex	
Male	22 (100)
Female	0
Homeless status	
Shelter	11 (50)
Street	7 (32)
Friends/family	4 (18)
Smoking	
Current or ex-smoker	15 (68)
Nonsmoker	3 (14)
Unknown	4 (18)
Alcohol abuse	
Yes	9 (41)
No	9 (41)
Unknown	4 (18)
Comorbidities	
None	12 (55)
HTN	5 (23)
DM	3 (14)
Heart disease or stroke	3 (14)
Anemia	2 (9)
Tuberculosis	1 (5)
Liver disease	1 (5)
Pathologic diagnosis	
NSCLC	16 (73)
ADC	6 (27)
SqCC	8 (36)
Adenosquamous	1 (5)
NSCLC, NOS	1 (5)
SCLC	3 (14)
Unknown	3 (14)
Stage	
IA	2 (9)
IB	2 (9)
IIA	1 (5)
IIIB	2 (9)
IV	15 (68)
Curative surgery	
Yes	4 (18)
No	18 (82)
Palliative surgery	
Yes	1 (5)
No	21 (96)
Chemotherapy	
Yes	4 (18)
No	18 (82)
Radiation therapy	
Yes	2 (9)
No	20 (91)
Hospitalization	
Yes	19 (87)
No	3 (14)

HTN, hypertension; DM, diabetes mellitus; NSCLC, non-small cell lung cancer; ADC, adenocarcinoma; SqCC, squamous cell carcinoma; NOS, not otherwise specified; SCLC, small cell lung cancer.

**Table 2:** Costs related to hospitalization and outpatient clinic visits (*n* = 14).

Subjects	Median	Range
Initial hospitalization		
Duration (days)	29	7–67
Total cost	8,619	2,295–27,839
Cost not covered by NHIS^a^	1,575	338–7,040
Outpatient visits		
Number of days	2	0–33
Total cost	949	19–5,916
Cost not covered by NHIS^a^	192	0–1,920
Further hospitalization		
Duration (days)	0	0–26
Total cost	0	0–6,435
Cost not covered by NHIS^a^	0	0–1,622
Total cost	12,513	4,280–27,858

^a^Costs that were not reimbursed by the National Health Insurance Service were paid by the homeless shelter or by the Seoul Metropolitan Government. Cost is stated in US dollars.

NHIS, the National Health Insurance Service.

**Table 3:** Cost comparison between early stage and advanced stage disease (*n* = 14).

Subjects	Early stage (*n* = 4)	Advanced stage (*n* = 10)
	Median (range)	Median (range)
Total cost	14,153 (8,299–20,283)	11,100 (4,280–27,858)
Admission		
Duration (days)	34 (10–46)	34 (18–67)
Cost	12,567 (6,700–20,142)	8,619 (4,233–27,839)
Room and board	2,674 (1,756–3,102)	2,466 (1,201–6,757)
Pharmacy	688 (639–852)	1,111 (295–6,052)
Laboratory	1,730 (1,536–3,801)	2,096 (545–6,917)
Radiation therapy	0 (0)	0 (0–1,418)
Operation/procedure	3,427 (184–4,994)	289 (0–4,947)
Others	4,090 (2,372–7,523)	2,613 (1,838–7,207)
Outpatient visits		
Number of visits	11 (3–17)	0 (0–33)
Cost	1,274 (140–2,221)	52 (0–5,916)

Cost is stated in US dollars. None of the comparisons reached statistical significance.
